# Development of a TaqMan-Probe-Based Multiplex Real-Time PCR for the Simultaneous Detection of Porcine Circovirus 2, 3, and 4 in East China from 2020 to 2022

**DOI:** 10.3390/vetsci10010029

**Published:** 2022-12-31

**Authors:** Jianwen Zou, Huaicheng Liu, Jing Chen, Jin Zhang, Xiaohan Li, Yunfeng Long, Yan Jiang, Wenliang Li, Bin Zhou

**Affiliations:** 1 MOE Joint International Research Laboratory of Animal Health and Food Safety, College of Veterinary Medicine, Nanjing Agricultural University, Nanjing 210095, China; 2 Animal, Plant and Food Inspection Center, Nanjing Customs, Nanjing 210019, China; 3 Institute of Veterinary Medicine, Jiangsu Academy of Agricultural Sciences, Nanjing 210014, China

**Keywords:** porcine circovirus (PCV), real-time qPCR, TaqMan probe, differential diagnosis, multiple infection

## Abstract

**Simple Summary:**

Porcine circovirus (PCV) is widespread in the world and causes huge economic losses to the pig industry. Recently, the infection rates of PCV2 and PCV3 have been increasing year by year in China, and a new type of virus, PCV4, discovered in 2019, was found to result in complex pathogenic infection. In this study, a TaqMan-probe-based multiplex real-time PCR (qPCR) assay was developed for the differential detection of PCV2, -3, and -4. A total of 535 clinical samples from Shandong, Zhejiang, Jiangsu, and Anhui provinces, East China, were included in the analyses to evaluate the application of multiplex qPCR. The resulting data showed that the individual positive rates of PCV2, PCV3, and PCV4 were 35.33%, 40.37%, and 33.08%, respectively; the mixed infection rates of PCV2 and PCV3, PCV2 and PCV4, and PCV3 and PCV4 were 31.03%, 30.09%, and 30.84%, respectively; the mixed infection rate of the three viruses was 28.22%. The developed assay showed extreme specificity, high sensitivity, and excellent reproducibility for the simultaneous detection and differentiation of three porcine circovirus types.

**Abstract:**

Porcine circovirus disease (PCVD) caused by porcine circovirus (PCV) is an important swine disease that is characterized by porcine dermatitis, nephropathy syndrome, and reproductive disorders in sows. However, disease caused by PCV2, PCV3, or PCV4 is hard to distinguish, so a rapid and sensitive detection method is urgently needed to differentiate these three types. In this study, four pairs of specific primers and the corresponding probes for PCV 2, -3, and -4, and porcine endogenous gene β-Actin as the positive internal reference index, were designed to establish a TaqMan multiplex real-time PCR (qPCR) assay for the simultaneous differential diagnosis of different types of viruses. The results showed that this assay has good specificity and no cross-reactivity with other important porcine viral pathogens. Furthermore, it has high sensitivity, with a detection limit of 101 copies/μL, and good reproducibility, with intra- and inter-group coefficients of variation below 2%. Subsequently, 535 clinical samples of suspected sow reproductive disorders collected from Shandong, Zhejiang, Anhui, and Jiangsu provinces from 2020 to 2022 were analyzed using the established assay. The results showed that the individual positive rates of PCV2, PCV3, and PCV4 were 31.03%, 30.09%, and 30.84%, respectively; the mixed infection rates of PCV2 and PCV3, PCV2 and PCV4, and PCV3 and PCV4 were 31.03%, 30.09%, and 30.84%, respectively; the mixed infection rate of PCV2, PCV3, and PCV4 was 28.22%. This indicated that this assay provides a convenient tool for the rapid detection and differentiation of PCV2, PCV3, and PCV4 in pig farms in East China. Our findings highlight that there are different types of porcine circovirus infection in pig farms in East China, which makes pig disease prevention and control difficult.

## 1. Introduction

Porcine circovirus disease (PCVD) is a general term for a variety of diseases caused by porcine circovirus (PCV) in pigs [1]. The PCV types that have been identified, including PCV1, PCV2, PCV3, and PCV4, are non-enveloped DNA viruses with a diameter of about 20 nm belonging to the genus Circovirus in the family Circoviridae [2]. The genome of PCV is a single-stranded, positive, circular DNA of about 1.7–2.0 kb in length, encoding structural protein capsid (Cap) and replicase (Rep) [3]. PCV1 is not pathogenic and is widely present in pigs; it also acts as a source of contamination of cell lines [4]. PCV2 was first discovered in 1998 and is the main pathogen of PCVD [5]. PCV2 currently has nine subtypes, among which PCV2d is the one currently prevalent in China [6]. PCV3 was discovered in 2016 and mainly causes porcine dermatitis and nephropathy syndrome (PDNS) [7]. PCV4 was discovered in 2019 and causes respiratory disease, diarrhea, and PDNS [8,9]. These viruses can lead to multisystem failure in weaning piglet syndrome, porcine dermatitis nephrotic syndrome, sow reproductive disorders, and other symptoms, causing a large number of sows to abort and piglets to die, followed by large economic losses for farms [10,11,12,13,14,15,16].

It is difficult to prevent and control porcine circovirus disease, because the three viruses are difficult to distinguish clinically [17,18,19], and there is no commercial vaccine nor detection method for them (PCV3 and PCV4), except for PCV2 [20]. Currently, the main detection method for PCV3 and PCV4 is real-time PCR [8,12], but repeated screening for PCV2, PCV3, and PCV4 is time consuming and laborious. False positives caused by sampling may occur due to the lack of internal reference. Therefore, it is necessary to establish a method that can detect these three pathogens simultaneously and with which false-positive results can be reduced.

Multiplex real-time PCR (multiplex qPCR) is a technique for detecting multiple genes in a single reaction by adding multiple pairs of specific primers and probes to a tube system; it is a fast and sensitive method [21]. In this study, we established a method that can detect PCV2, PCV3, and PCV4 simultaneously. This method effectively distinguishes these three pathogens; meanwhile, the introduction of internal reference can effectively eliminate false positives caused by sampling failure. It provides a fast, sensitive, and specific tool for distinguishing pathogens in pig farms.

## 2. Materials and Methods

### 2.1. Primers and Probes

Based on the conserved region sequences of the PCV2 Cap gene (KT719404.1), the PCV3 Rep gene (MK580468.1), and the Cap gene of PCV4 (MT311854.1), three pairs of specific primers were designed. Primers were used for the construction of recombinant standard plasmids and the amplification of real-time PCR, respectively. The primers and probes for the corresponding porcine-derived β-Actin were previously described by Wang et al [22]. All the sequences of special primers and probes synthesized by Genscript Biotech Co., Ltd. (Nanjing, China) are shown in Table 1.

### 2.2. Viruses, Nucleic Acid, and Clinical Samples

JEV strain NJ-2008 (GenBank accession No. GQ918133), CSFV strain Shimen (GenBank accession No. AF092448), and clinical samples positive for PCV2, PCV3, PRV, PRRSV, and PDCoV, as confirmed with a PCR assay, were stored in the lab. PCV1-positive nucleic acid was a gift provided by Prof. Song Gao (Yanzhou University, Yangzhou, China). ASFV-positive nucleic acid was a gift provided by Dr. Xiaowen Li (New Hope Liuhe Co., Ltd., Qingdao, China). TGEV-PEDV vaccine (WH-1R + AJ1102-R) was purchased from Wuhan Keqian Biological Co. (Wuhan, China). The above viruses and positive nucleic acids were used for plasmid standard construction and specificity tests in this study. The plasmid of the PCV4 Cap gene (MT311854.1), named pUC21-PCV4, was synthesized by Tsingke Biotechnology Co., Ltd. (Beijing, China). A total of 535 clinical samples, including lymph nodes, spleens, and blood from pigs with respiratory and/or reproductive problems accompanied by progressive weight loss, were collected in Shandong, Zhejiang, Anhui, and Jiangsu from 2020 to 2022 and kept in the lab.

### 2.3. DNA/RNA Extraction and Reverse Transcription

All samples were resuspended in 0.9% stroke-physiological saline solution and homogenized. RNA/DNA was extracted from 150 μL homogenized samples using Biomiga^®^ DNA/RNA Multiprep Kit following the manufacturer’s instructions (Biomiga, Inc., San Diego, CA, USA). TransScript^®^ II Reverse Transcriptase Kit was used to reverse-transcribe the extracted RNA into cDNA according to the manufacturer’s instructions. DNA and cDNA products were stored at −40 °C. The DNA of the clinical samples was used as the template for multiple qPCR, and the DNA and cDNA of viruses were used in the specificity tests.

### 2.4. Construction of Recombinant Plasmids

The conservative region of the PCV2 Cap gene or PCV3 Rep gene, respectively, was cloned into a pEasy vector. In order to obtain a large number of positive plasmids, the constructed plasmids were subjected to positive transformation into Trans-T1 competent cells. Then, the bacterial cultures were shaken and grown for 12–16 h at 37 °C. The plasmids were extracted using Omega EZNA Plasmid Mini Kit I and sent for DNA sequencing. Two recombinant plasmids, pEasy-PCV2 and pEasy-PCV3, were used as the standard plasmids to determine the concentration using a NanoDrop spectrophotometer (Thermo Fisher, Waltham, MA, USA).

### 2.5. Optimization of Multiplex qPCR Assay

The standard plasmids were 10-fold-diluted from 3 × 10^7^ to 3 × 10^4^ copies/μL as amplification templates. Alternatively, the three diluted standard plasmids were mixed in equal proportions to ensure that the concentration of the mixed standard plasmids was up to 1 × 10^4^ copies/μL. Four pairs of the primers and probes at a concentration of 10 μM were added to one reaction system for simultaneous detection. Different concentrations of probes and primers were set to explore the optimal reaction conditions for this multiplex reaction. The reaction procedure was 180 s of pre-denaturation at 95 °C for 40 cycles, with 10 s of denaturation at 95 °C, 10 s of annealing at 60 °C, and 20 s of extension at 72 °C. The fluorescence channels of the qPCR instrument were set as follows: channel 1, FAM; channel 2, HEX; channel 3, Texas Red; and channel 4, Cy5. Fluorescence signals were also collected with a Mic PCR real-time PCR instrument (IDEXX, Westbrook, ME, USA).

### 2.6. Establishment of Standard Curves

Using the optimal reaction system as a benchmark, three recombinant standard plasmids were 10-fold-diluted from 10^7^ to 10^1^ copies/μL, and seven concentrations of recombinant standard plasmids were used as templates for real-time PCR. The standard curves were plotted using Prism software.

### 2.7. Specificity of Multiplex qPCR Assay

The three constructed standard plasmids were used as positive controls, and the DNA or cDNA of ASFV, PRRSV, CSFV, PRV, PEDV, TGEV, PCV1, and PDCoV extracted from the viruses or positive disease material was used as template. ddH_2_O was used as a negative control, and the specificity was verified with real-time PCR amplification in the optimal reaction system.

### 2.8. Sensitivity of Multiplex qPCR Assay

The standard plasmids of PCV2, PCV3, and PCV4 were 10-fold-diluted, and the plasmids of the corresponding concentration gradients were mixed. After mixing, multiplex real-time PCR amplification was performed in the optimal reaction system, and three replicate experiments were performed for each concentration to confirm the detection limits of this multiplex real-time PCR assay.

### 2.9. Repeatability of Multiplex qPCR Assay

The standard plasmids of PCV2, PCV3, and PCV4 were diluted from 10^7^ copies/μL to 10^4^ copies/μL in a 10-fold ratio; then, the plasmids of the corresponding concentration gradients were mixed, and the mixture was subjected to multiplex real-time PCR amplification in the optimal reaction system. Three replicates were made for each concentration gradient, and assays were performed every other week. The different groups of corresponding concentrations were compared, and their intra-group and inter-group coefficients of variation were calculated to verify their reproducibility.

### 2.10. Clinical Sample Detection

The 535 clinical samples (tissues and blood) kept in the lab were registered. Nucleic acid extraction was performed using a nucleic acid extraction kit. The DNA of the clinical samples was used as the template; standard plasmids were used as positive controls; and ddH_2_O was used as a negative control. Multiplexed fluorescent quantitative PCR amplification was performed using the optimized optimal reaction system and reaction procedure to identify the positive rate of each pathogen.

## 3. Results

### 3.1. Optimization of qPCR Reaction Conditions

Among the four fluorophores used in the multiplex detection method, the fluorescence of Cy5 was very susceptible to interference from other fluorophores. Therefore, the main purpose of the reaction optimization system was to improve the performance of the Cy5 fluorophores. As shown in Table 2, it was concluded that the best final concentrations of probes and primers are 600 nM and 1200 nM, respectively.

The qPCR reaction system was as follows: 20 μL of 2× AceQ qPCR Probe Master Mix (Vazyme Biotech Co., Ltd.); initial concentrations of 10 μM PCV2-F, PCV2-R, PCV3-F, PCV3-R, PCV4-F, PCV4-R, ACTB-F, and ACTB-R primers of 0.6 μL each; initial concentrations of 10 μM PCV2-Probe, PCV3-Probe, PCV4-Probe, and ACTB-Probe probes of 0.3 μL each; volumes of DNA template and ddH_2_O of 4 μL; total volume of the system of 20 μL. The PCR reaction program was as follows: 95 °C pre-denaturation for 3 min; then, 40 cycles included denaturation at 95 °C for 10 s, annealing at 60 °C for 10 s, and extension at 72 °C for 20 s. After optimization, the fluorescence channels of the qPCR instrument were set as follows: channel 1, FAM; channel 2, HEX; channel 3, CY5; and channel 4, Texas Red. This experiment employed a Mic PCR real-time PCR instrument to collect fluorescence signals.

### 3.2. Establishment of Standard Curves

**Figure 1:**
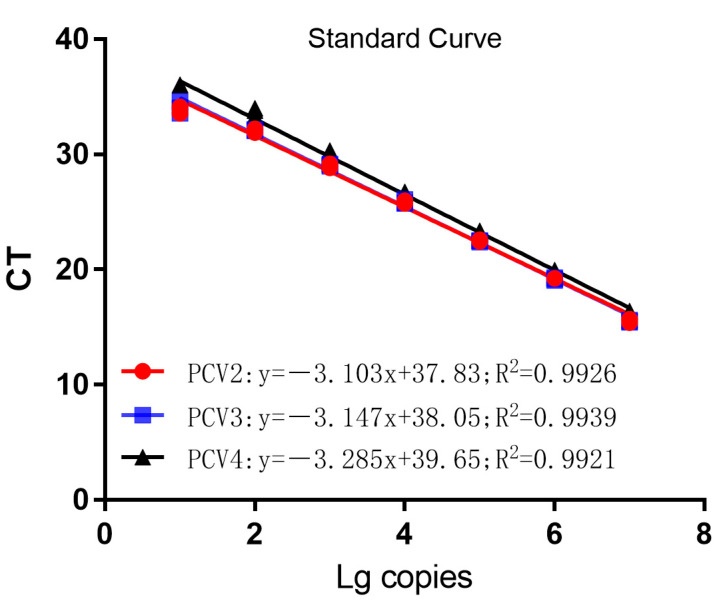
Standard curves of multiplex TaqMan qPCR.

The three recombinant standard plasmids, diluted to 10^7^–10^1^ copies/μL in a 10-fold gradient, were amplified using single TaqMan fluorescent quantitative PCR according to the optimal reaction system and reaction procedure. The standard curve was established using the obtained CT values as the vertical coordinates and the logarithm of the concentration of the plasmid as the horizontal coordinate. As shown in Figure 1, the correlation coefficients of R^2^ = 0.9926 for PCV2, R^2^ = 0.9939 for PCV3, and R^2^ = 0.9921 for PCV4 demonstrated that the amplification curves of the three recombinant plasmids constructed in this experiment had a good linear relationship with the CT values.

### 3.3. Specificity of Multiplex qPCR Assay

**Figure 2:**
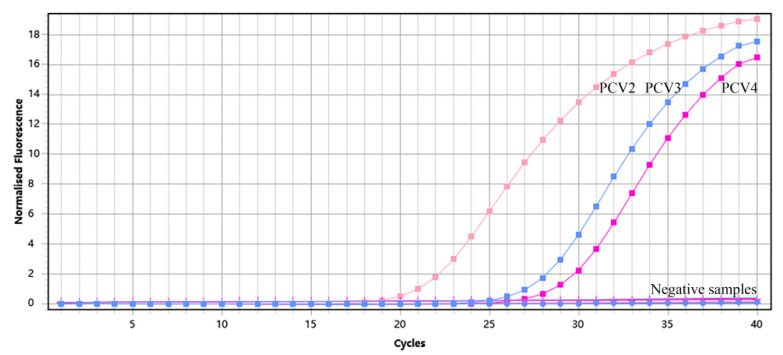
Amplified curves of specificity analysis of multiplex TaqMan qPCR.

The plasmids of PCV2, PCV3, and PCV4 were used as positive controls; DNA/cDNA extracted from PCV1-, ASFV-, CSFV-, PRRSV-, PRV-, PEDV-, TGEV-, JEV-, and PDCoV-positive material were used as templates; and ddH_2_O was used as a negative control. TaqMan qPCR was performed in the optimal reaction system for fluorescence quantitative PCR amplification. The results showed that only PCV2, PCV3, or PCV4 nucleic acid from the positive samples was detected using this assay (Figure 2). However, the DNA and cDNA of other viral pathogens, such as ASFV, PRRSV, CSFV, and PRV, were not amplified (Table 3). Meanwhile, we found that no false positives were found in any of the samples, based on the amplification of β-Actin. Together, these data indicate that this method has good specificity.

### 3.4. Sensitivity of Multiplex qPCR Assay

**Figure 3:**
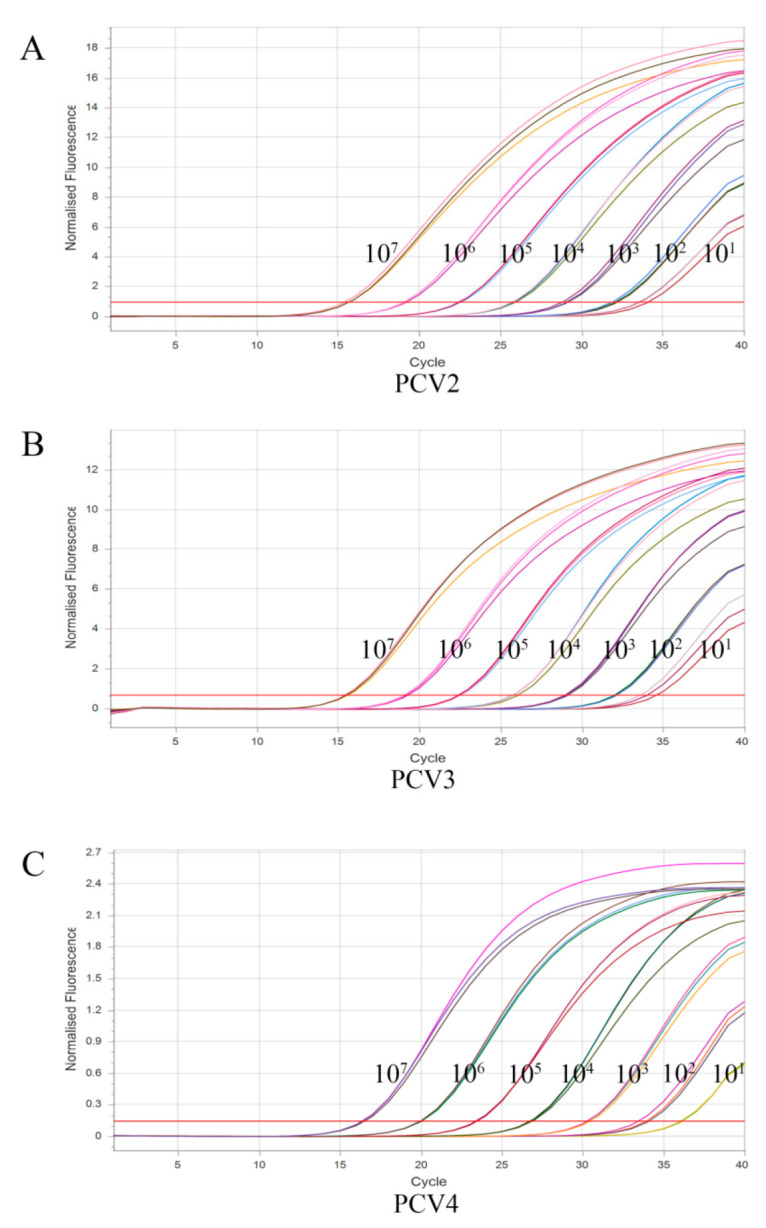
Sensitivity analysis of multiplex TaqMan qPCR. (**A**), sensitivity analysis of PCV2; (**B**), sensitivity analysis of PCV3; (**C**), sensitivity analysis of PCV4.

The sensitivity of the assay was investigated using the optimal reaction system for PCV2, PCV3, and PCV4 at seven concentrations ranging from 1 × 10^7^ copies/μL to 1 × 10^1^ copies/μL. As shown in Figure 3, the detection limits of the assay were 10^1^ copies/μL for PCV2, PCV3, and PCV4, suggesting that the assay has good sensitivity.

### 3.5. Repeatability of Multiplex Real-Time PCR Assay

The repeatability and reproducibility of the developed multiplex TaqMan qPCR assay were evaluated using the recombinant standard plasmids, which were 10-fold-diluted from 10^7^ copies/μL to 10^4^ copies/μL and used as templates. As shown in Table 4, the results showed that the coefficients of variation (CVs) of CT values in both intra-group reproducibility experiments and inter-group reproducibility experiments were from 0.09% to 0.80% and from 0.16% to 1.94%, respectively, indicating that the assay has good reproducibility.

### 3.6. Detection of Clinical Samples

A total of 535 samples, including clinical tissue samples and blood samples, were collected from various breeding sites in Shandong, Zhejiang, Anhui, and Jiangsu provinces, from 2020 to 2022. The DNA of PCV2, PCV3, and PCV4 in the 535 clinical samples was detected using the established method. As shown in Table 5, the results showed that the infection rates of PCV2, PCV3, and PCV4 were 35.33% (189/535), 40.37% (216/535), and 33.08% (177/535). The co-infection rates of PCV2 and PCV3, PCV2 and PCV4, and PCV3 and PCV4 were 31.03% (166/535), 30.09% (161/535), and 30.84% (165/535). The mixed infection rate of PCV2/-3/-4 was 28.22% (151/535). All samples were tested using the specific β-Actin primers and probe and showed positive data, proving that all samples had been sampled normally, and no false positives were observed.

## 4. Discussion

PCVD is a general term for a variety of diseases in pigs caused by PCV that mainly manifests as PMWS and reproductive disorders in sows and that is the most harmful to both sows and piglets [9,13,23,24]. Since the discovery of PCV3 in 2016, PCV3 has also gradually shown its harmful effects on sows and piglets [11,17,25,26,27], and the discovery of PCV4 in 2019 has further complicated the etiology of circovirus disease [19,28,29,30,31]. Therefore, farmers in China’s pig industry have come to believe that mixed infections of PCV2, PCV3, and PCV4 have now become the norm [18,32]. In this study, we developed a multiplex TaqMan qPCR assay for the differential diagnosis of PCV2, PCV3, and PCV4 that can identify the causative agent of circovirus disease, thus enabling appropriate treatment to be carried out. Meanwhile, to ensure accurate test results, the amplification of the β-Actin gene is performed, which ensures the accuracy and reliability of the results. In this assay, four pairs of primer–probe sets can be added to one tube system, thus allowing three pathogens to be simultaneously detected in one reaction. Remarkably, the assay has good specificity and no cross reactivity with common viral pathogens. PCV at 10^1^ copies/μL can be detected using this assay, with a coefficient of variation below 2%, suggesting good sensitivity and reproducibility. Therefore, the detection assay we established provides a powerful tool for the effective and rapid diagnosis of mixed infection with different PCV types in the field.

Recently, 2707 serum samples of pigs were randomly collected from 17 provinces in China between September 2018 and March 2022 and were analyzed with PCR assays. PCV molecular epidemiological survey data also showed that PCV3 infection was prevalent in the overall population, with 31.07% and 100.0% at the sample and province levels, respectively [33], suggesting that PCV3 infection has a widespread distribution in China. Another report showed the prevalence of PCV1/-2/-3 in wild boar in Jiangxi province. Of 138 wild boar samples, the positive rates of PCV1, PCV2, and PCV3 were 21.7%, 22.5%, and 5.8%, respectively [34]. Consistent with our data, the above study showed that the positive rates of PCV1 and PCV2, and PCV2 and PC3 co-infections were 7.3% and 3.6%. Interestingly, a PCV epidemiological study in Southwest China showed high prevalence rates of PCV2 and PCV3 (26.46% and 33.46%, respectively). Notably, the coinfection rate doubled from 2020 (5.75%) to 2022 (10.45%) [35]. Strikingly, in this study, we used this method to distinguish PCV types in pig samples collected from four provinces (Shandong, Jiangsu, Anhui, and Zhejiang) in East China. The resulting data indicated that PCV infections are common in China and that mixed infections with different types of PCV are much more common than individual PCV infections alone. It should be noted that, in the retrospective testing of samples of some severe abortions in pig farms with prevalence of the PRRSV NADC30 strain, mixed PCV infection was reported to be extremely high, which also illustrates the complex etiology of current sow reproductive disorders. The frequent presentation of multiplex infections creates new obstacles to disease prevention and control in pig farms [36].

Although commercial PCV2 vaccines exist, PCV infection is still common in farms due to the iteration of PCV2 epidemic subtypes and the existence of PCV3 and PCV4. Mixed infection of PCV with other sow reproductive disorders has become the norm [37,38]. Therefore, it is necessary to conduct regular examinations for prevention and control in pigs. The established multiplex real-time PCR assay for PCV2, PCV3, and PCV4 has good sensitivity, specificity, and reproducibility, and it provides a rapid and convenient tool with clinical application in the screening of the pathogens of circovirus disease in primary farms.

## 5. Conclusions

A multiplex TaqMan real-time PCR assay capable of detecting PCV2, PCV3, and PCV4 simultaneously was successfully established in this study. The method has good specificity, sensitivity, and reproducibility, and it has no cross-reactivity with common pathogens. The PCV epidemiological survey data from four provinces in East China showed that PCV is widespread in these areas. These data remind Chinese pig farmers that PCV infection is becoming increasingly serious and pave the way for the establishment of preventive measures.

## Figures and Tables

**Table 1:** Primers and probes used in this study.

Pathogen	Primers and Probes	Sequence (5′ end to 3′ End)	Length (bp)	Gene	Position
PCV2	PCV2-F	TTACACGGATATTGTATTCCTGGTCG	295	Cap	1089–1383 ^a^
	PCV2-R	GTGGGCTCCAGTGCTGTTATTCTA			
	PCV2-QF	AGTCTCAGCCACAGCTGATT	128		1175–1302 ^a^
	PCV2-QR	TCCTCCCGCCATACCAT			
	PCV2-Probe	Cy5-AGCCCTTCTCCTACCACTCCCGCT-BHQ2			
PCV3	PCV3-F	ACAAAGAGGCCAGCGAGTA	381	Rep	470–850 ^b^
	PCV3-R	CATCCAGAATAACAGCACCC			
	PCV3-QF	CGGATTCTGACGGAGACG	125		568–692 ^b^
	PCV3-QR	TCACGCGGTTTACCCAACCC			
	PCV3-Probe	FAM-GCTATGGGCGGGGTTTGCGT-TAMRA			
PCV4	PCV4-QF	GTCCACACCTGCACAAAGTT	120	Cap	1114–1233 ^c^
	PCV4-QR	CCTCCACTTCCAGCCTAACA			
	PCV4-Probe	Texas Red-AGGTCCTGGTCCGCCATGCT-BHQ2			
β-Actin	ACTB-QF	CCCTGGAGAAGAGCTACGAG	175	Wang [22]	
	ACTB-QR	AGGTCCTTCCTGATGTCCAC			
	ACTB-Probe	HEX-CGGCAACGAGCGCTTCCGGT-BHQ1			

^a^ GenBank accession No. KT719404.1; ^b^ GenBank accession No. MK580468.1; ^c^ GenBank accession No. MT311854.1.

**Table 2:** Optimization of primer and probe concentrations. CT values of PCV2, PCV3, and PCV4 detected using multiplex TaqMan qPCR with different concentrations of probes and primers.

PCV2 (CY5)						
Probe concentration (nM)	Primer concentration (nM)					
	200	400	600	800	1000	1200
200	25.79	25.81	25.66	25.71	25.96	25.63
400	26.05	25.54	24.96	25.62	25.25	25.77
600	25.76	25.80	25.99	24.79	24.75	24.66
800	25.41	25.31	25.42	25.87	25.23	25.60
1000	26.07	25.81	25.53	25.45	25.23	25.41
PCV3 (FAM)						
Probe concentration (nM)	Primer concentration (nM)					
	200	400	600	800	1000	1200
200	28.33	28.68	28.75	28.59	28.66	28.91
400	28.76	28.61	28.66	28.67	28.69	28.51
600	28.73	28.92	28.69	28.53	27.96	27.88
800	29.00	28.22	28.52	29.00	28.93	27.95
1000	28.29	27.43	28.80	28.92	28.43	28.12
PCV4 (Texas Red)						
Probe concentration (nM)	Primer concentration (nM)					
	200	400	600	800	1000	1200
200	29.01	29.17	29.27	29.15	29.00	29.17
400	29.07	29.00	28.75	28.60	28.78	28.65
600	28.94	28.27	28.40	28.34	28.28	28.25
800	28.95	27.83	28.18	28.08	28.11	28.00
1000	27.67	27.47	27.51	27.61	27.66	27.54

**Table 3:** Ct values of specificity analysis of multiplex TaqMan qPCR.

Sample Number	Pathogen	Ct Value ^a^		
		FAM	Cy5	Texas Red
1	PCV2	21.39 ± 0.11	-	-
2	PCV3	-	26.34 ± 0.15	-
3	PCV4	-	-	29.04 ± 0.12
4	PCV1	-	-	-
5	ASFV	-	-	-
6	PRV	-	-	-
7	CSFV	-	-	-
8	JEV	-	-	-
9	PDCoV	-	-	-
10	PRRSV	-	-	-
11	TGEV	-	-	-
12	PEDV	-	-	-

^a^ Data are means ± S.D. from three independent experiments.

**Table 4:** Repeatability and reproducibility analyses of multiplex TaqMan qPCR.

Standard Plasmid	Concentration (Copies/μL)	Intra-Assay			Inter-Assay		
		Mean	S.D.	CV (%)	Mean	S.D.	CV (%)
PCV2	10^7^	15.24	0.102	0.67	15.28	0.061	0.40
	10^6^	19.24	0.094	0.49	19.24	0.061	0.32
	10^5^	23.52	0.188	0.80	23.56	0.038	0.16
	10^4^	26.13	0.037	0.14	26.29	0.226	0.86
PCV3	10^7^	15.24	0.102	0.67	15.31	0.103	0.67
	10^6^	19.22	0.083	0.43	19.25	0.046	0.24
	10^5^	23.54	0.184	0.78	23.71	0.247	1.04
	10^4^	26.11	0.024	0.09	26.34	0.308	1.17
PCV4	10^7^	15.21	0.063	0.42	15.28	0.052	0.34
	10^6^	19.15	0.026	0.14	19.17	0.073	0.38
	10^5^	24.29	0.107	0.44	24.43	0.140	0.57
	10^4^	26.44	0.042	0.16	26.90	0.522	1.94

**Table 5:** Analysis of clinical samples using multiplex TaqMan qPCR.

Pathogen Type	Positive Samples ^a^	Infection Rate (%)
PCV2	189	35.33
PCV3	216	40.37
PCV4	177	33.08
PCV2 + PCV3	166	31.03
PCV2 + PCV4	161	30.09
PCV3 + PCV4	165	30.84
PCV2 + PCV3 + PCV4	151	28.22
β-Actin	535	100
In total	535	/

^a^ These data are representative of three independent experiments.

## Data Availability

Not applicable.
